# Long-term outcomes and patterns of failure after empiric SBRT for presumed early-stage lung tumors

**DOI:** 10.3389/fonc.2025.1705311

**Published:** 2025-11-25

**Authors:** Karim El-Marouk, Esra Degerli, Lukas Käsmann, Sophie Kröninger, Diego Kauffmann-Guerrero, Amanda Tufman, Niels Reinmuth, Thomas Duell, Farkhad Manapov, Claus Belka, Chukwuka Eze, Sina Mansoorian

**Affiliations:** 1 Department of Radiation Oncology, University Hospital, Ludwig Maximilian University of Munich LMU Munich, Munich, Germany; 2 German Cancer Consortium (DKTK), Partner Site Munich and German Cancer Research Center (DKFZ), Heidelberg, Germany; 3 German Center for Lung Research (DZL), Comprehensive Pneumology Center Munich (CPC-M), Munich, Germany; 4 Department of Medicine V-Pneumology, University Hospital, Ludwig-Maximilians-University Hospital (LMU) Munich, Munich, Germany; 5 Bavarian Cancer Research Center (BZKF), Munich, Germany; 6 Asklepios Kliniken GmbH, Asklepios Fachkliniken Munich, Gauting, Germany; 7 RADIO-LOG Medical Care Centre for Radiation Therapy, Günzburg, Germany

**Keywords:** stereotactic body radiation therapy, empiric SBRT, early-stage non-small cell lung cancer, presumed lung cancer, local control, recurrence patterns, distant metastasis

## Abstract

**Background:**

Stereotactic body radiation therapy (SBRT) is the established standard of care for medically inoperable early-stage non-small cell lung cancer (ES-NSCLC). When a biopsy is unfeasible, it is often delivered empirically, yet long-term outcomes and failure patterns remain underreported.

**Methods:**

A total of 56 patients with clinically staged T1–T3N0M0 lung tumors treated with empiric SBRT (2011–2022) were retrospectively analyzed. Nineteen patients with recurrence were assessed for failure patterns and survival. Overall survival (OS), progression-free survival (PFS), local failure-free survival (LFFS), regional failure-free survival (RFFS), and distant metastasis-free survival (DMFS) were estimated using the Kaplan–Meier method. Competing risk analysis was performed with death treated as a competing event.

**Results:**

At a median follow-up of 80.4 months (95% CI: 65.2–95.6), 17 patients (30.4%) were alive. Median PFS and OS were 28.5 months (95% CI: 16.4–40.8) and 41.7 months (95% CI: 14.0–69.4), respectively. LFFS was 84.1% at 5 years and 67.3% at 10 years, RFFS was 64.7% at 5 years and 58.8% at 10 years, and DMFS was 62.4% at 5 years and 56.1% at 10 years. Pathologic confirmation of recurrence was obtained in 10 patients, identifying NSCLC in six, small cell lung cancer in three, and urothelial carcinoma in one. Local failures were infrequent and occurred early (median 8.3 months), whereas regional and distant recurrences occurred later (median 13.5 and 22.8 months). At 10 years, the estimated cumulative incidence function was 6.3% for local failure, 14.8% for regional failure, 16.6% for distant failure, and 55.8% for death.

**Conclusion:**

Empiric SBRT provides durable local control in presumed early-stage NSCLC, but outcomes are limited by comorbid mortality and systemic progression. These findings emphasize its effectiveness as a local therapy and the need for prolonged surveillance and systemic strategies.

## Introduction

Stereotactic body radiation therapy (SBRT) has become a standard treatment option for patients with inoperable early-stage non-small cell lung cancer (NSCLC), demonstrating excellent local control and survival outcomes (1–6). Emerging evidence suggests that, in selected cases, SBRT may offer outcomes comparable to those of surgical resection (7–9). However, patients with pathologically confirmed disease (pTNM staging) generally experience better outcomes than those staged clinically (cTNM), emphasizing the importance of histological confirmation (10). Histopathological examination plays a central role in guiding treatment decisions and assessing prognosis in NSCLC. Beyond confirming malignancy, it enables molecular profiling, which can identify therapeutic targets and provide important prognostic insights (11–13). Furthermore, histological subtype can influence surgical planning and determine the extent of lung resection required (14).

Guidelines recommend either surgical resection or tissue biopsy for pulmonary lesions that are highly suspicious for malignancy (15). Transbronchial biopsy offers a sensitivity ranging from 50% to 91%, depending on lesion size and location (16, 17). Transthoracic needle biopsy generally provides higher diagnostic accuracy, with sensitivity exceeding 90% regardless of tumor size (18). Despite these options, there are instances in clinical practice where biopsy results remain inconclusive or cannot be obtained due to patient frailty, comorbidities, or procedural risk. In such cases, empiric SBRT may be pursued based on clinical and radiological assessment alone. Several studies have demonstrated comparable outcomes for patients treated with or without pathologic confirmation (19–21). Nonetheless, recurrence patterns in this empirically treated population remain poorly defined, particularly in the absence of histological data that could inform on tumor behavior.

Given these diagnostic and therapeutic considerations, we conducted a retrospective single-center analysis to evaluate recurrence dynamics and clinical outcomes following empiric SBRT in patients with clinically diagnosed early-stage NSCLC. Our objective was to identify failure patterns and characterize post-treatment trajectories in this unique and understudied cohort.

## Patients and methods

In a previous analysis, we reported outcomes and toxicities for 61 lung lesions treated with SBRT between 2011 and 2022. These lesions were suspected to be clinically staged T1–T3N0M0 lung tumors based on the Union for International Cancer Control (UICC) 8th edition, without histopathological confirmation. Patient inclusion and exclusion criteria, along with baseline characteristics, are described in detail in our previous publication (22). The decision to proceed with SBRT according to the Swensen criteria (23) was made by a multidisciplinary thoracic tumor board. This risk assessment primarily utilized the Solitary Pulmonary Nodule (SPN) Malignancy Risk Score (Mayo Clinic Model). This model evaluates six independent variables related to patient demographics and nodule morphology: patient age, smoking history, nodule size and spiculation, the presence of a prior extra thoracic malignancy diagnosed more than 5 years earlier, lesion location in the upper lobe, and metabolic activity on 18F-FDG PET/CT. Based on the calculated probability, patients were classi!ed into three commonly used clinical risk groups: low (<5%), intermediate (5%–60%), and high (>60%) (23).

For the current study, we extended the follow-up period through August 2025, focusing specifically on patients who experienced any form of recurrence. Unlike our previous lesion-level analysis, this study evaluated outcomes at the patient level.

**Figure 1:**
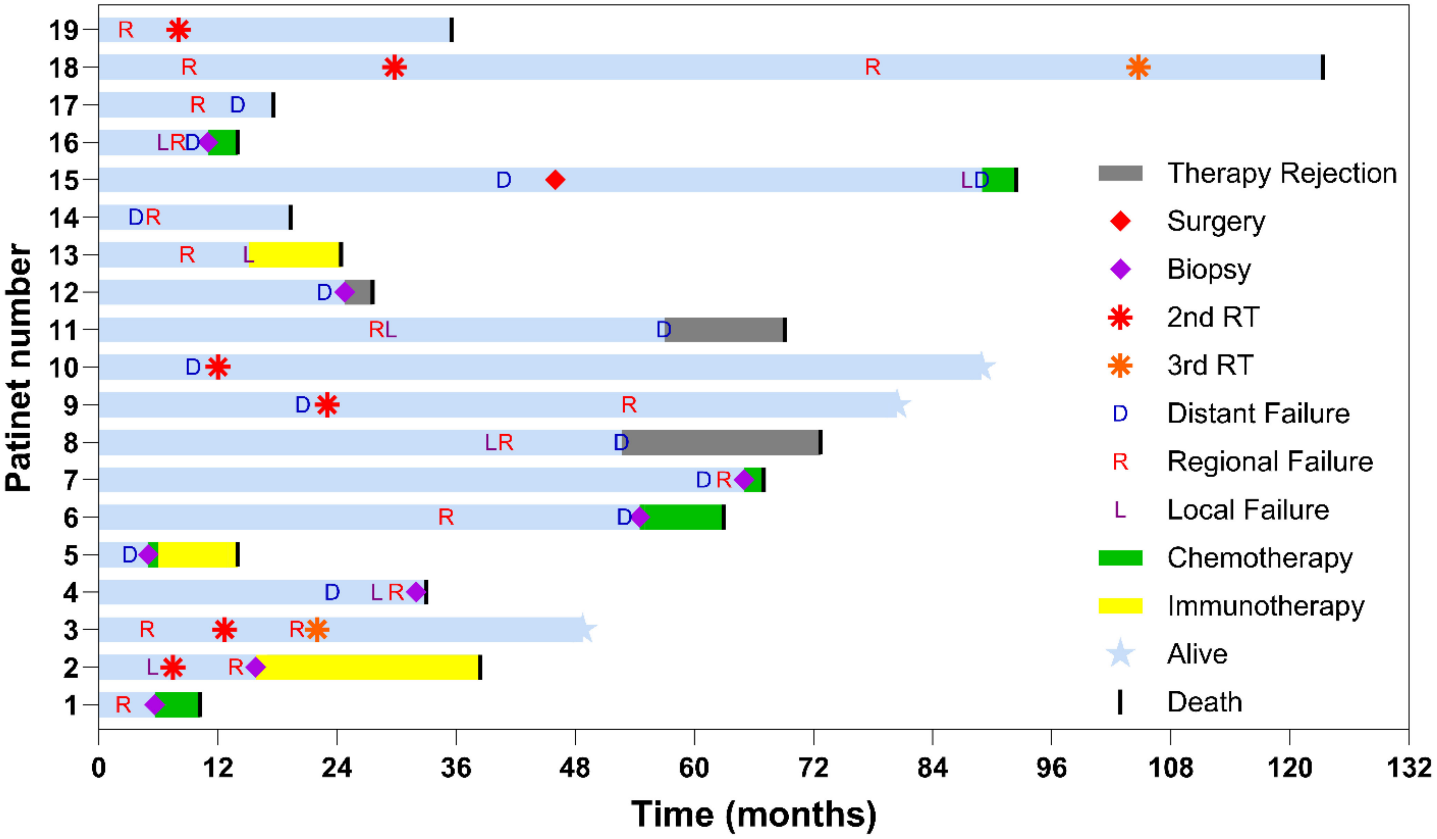
Detailed history of patients with any recurrence after stereotactic body radiation therapy (SBRT). The plot depicts the clinical course of 19 patients who experienced treatment failure after SBRT. Each horizontal bar represents the timeline for a single patient, measured in months from the completion of the first radiotherapy. The length of each blue bar shows the follow-up duration for each patient, with stars indicating patients still alive at the last follow-up. Several patients experienced multiple types of failure (local, regional, and distant) and sequential interventions, such as surgery or systemic therapy.

A total of 19 patients developed recurrence. We analyzed the behavior and progression patterns of these tumors, with emphasis on recurrence patterns and patient outcomes following additional lines of therapy. To illustrate the course of disease and treatment, personalized patient timelines are shown using a swimmer plot (**Figure 1**).

Tumor response was assessed according to the Response Evaluation Criteria in Solid Tumors (RECIST), version 1.1 (24). Local failure was defined as recurrence within the high-dose region of the irradiated field. Regional failure was defined as newly involved regional lymph nodes (hilar, mediastinal, or supraclavicular) or new pulmonary lesions in the ipsilateral lung. Distant metastasis was defined as new lesions in the contralateral lung, pleural metastases, or distant organ or lymph node involvement.

In the present report, overall survival (OS) was calculated from the completion of SBRT to death from any cause. Local failure-free survival (LFFS), regional failure-free survival (RFFS), and distant metastasis-free survival (DMFS) were measured from the completion of SBRT to the last follow-up with available staging imaging information. Progression-free survival (PFS) was defined as the time from the completion of SBRT to the first documented failure or death, whichever occurred first. All survival outcomes were estimated using the Kaplan–Meier method. The median follow-up was calculated using the reverse Kaplan–Meier method. Survival comparisons across variables, e.g., gross tumor volume (GTV) dose, were assessed using the log-rank test. To analyze recurrence patterns, competing incidence functions that account for death were used as a competing risk.

An exploratory analysis was performed to assess potential associations between biologically effective dose (BED) and relapse patterns, including local, regional, and distant failures. BED values were dichotomized using the median split. Stratified analyses were then conducted to examine possible predictors of OS, PFS, LFFS, RFFS, and DMFS based on GTV, BED, age, and tumor diameter. All comparisons were made using the log-rank test.

The BED was calculated to compare radiation impact across different fractionation schedules using the formula BED = n × d × [1 + d/(α/β)], where n is the number of fractions, d is the dose per fraction, and α/β = 10 Gy for tumor tissue. This metric enables the comparison of treatment intensity across regimens.

This retrospective analysis was conducted with approval from the institutional ethics committee (ID: 17-230). Informed consent for treatment and data use was obtained from all patients at the time of treatment. All statistical analyses were performed using IBM SPSS Statistics version 29.0.1, R version 4.4.2 (via RStudio 2024.12.1 Build 563), and GraphPad Prism version 10.5.0 (GraphPad Software, Boston, MA, USA).

## Results

### Patients and treatment characteristics

For all other patient characteristics, please refer to our previous results (22). Briefly, the median age at the time of SBRT was 69 years (range, 57–88), and the median Charlson Comorbidity Index (CCI) was 5 (range 2–13). Among smokers, the median tobacco consumption was 50 pack-years (py) (range, 5–120). The median forced expiratory volume in 1 second (FEV_1_) was 1.4 L (range, 0.5–2.3 L), the maximum forced vital capacity (FVC_max_;) was 76% of the predicted value (range, 26%–117%), and the diffusing capacity of the lung for carbon monoxide (DLCO) was 41% of the predicted value (range, 15%–109%). Prior to SBRT, biopsy procedures were attempted in 29 patients (51.8%); however, the results did not yield a definitive histological diagnosis.

The median GTV was 5.5 cc [interquartile range (IQR), 2.3–10.2 cc]. The median biologically effective dose, assuming an α/β ratio of 10 (BED_10_), was 95.2 Gy (IQR, 95.2–105.0 Gy), and the median BED_10_ maximum dose (D_max_) was 133.3 Gy (IQR, 131.3–146.4 Gy).

Individual characteristics of all 19 patients with recurrence—including tumor size, radiation dose and fractionation, and treatment outcomes—are summarized in **Tables 1A, B** and **Figure 1**.

**Table 1A:** Individualized characteristics and outcome of all patients with any recurrence.

Patient no.	Tumor volume [cc]	Dose fractionation	Time to LF (mo)		Time to RF (mo)	Location RF	Time to DF (mo)	DF location	Histology after recurrence	Systemic therapy after recurrence	OS (mo)
1	6.2	3 × 15 Gy at 65%	–	2.5		N1	–	–	SCLC	Carboplatin and etoposide	10.2
2*	8.4	1 × 30 Gy at 80%	5.5	13.8		N1	–	–	SCC	Pembrolizumab	38.4
3*	5.6	3 × 15 Gy at 65%	–	4.9		N3 (SCV)	–	–	–	–	48.8
4	1.2	3 × 13.5 Gy at 65%	30.7	30.7		N1	23.6	Bone	ACC	–	33.0
5	1.5	8 × 7 Gy at 70%	–	–		–	3.2	Liver, bone (m)	UC*	Carboplatin and paclitaxel	13.9
6	0.5	3 × 13.5 Gy at 65%	–	35.0		N1, ipsi. lung	53.1	Liver, soft tissue	LCC	Carboplatin and paclitaxel	62.7
7	1.7	3 × 13.5 Gy at 65%	–	64.2		N2 (m)	64.2	Bone (m)	SCLC	Carboplatin	65.2
8	3.0	8 × 7 Gy at 70%	39.5	39.5		N1, 2, 3	52.7	Bone (m)	–	–	72.7
9*	3.5	3 × 13.5 Gy at 65%	–	53.5		Ipsi. lung	20.6	Contr. lung	–	–	80.4
10*	1.8	8 × 7.5 Gy at 80%	–	–		Ipsi. lung	9.6	Contr. lung	–	–	88.9
11	4.1	9 × 5 Gy at 100%	29.2	28.5		Ipsi. lung	57.0	Pleu., CNS	–	Rejected	69.2
12	36.8	8 × 7.5 Gy at 80%	–	–		–	22.8	Contr. lung, skin, CNS, liver, peritoneal	LCC	Rejected	27.6
13**	122.9	10 × 5 Gy at 100%	15.2	8.9		N2 (m)		Contr. lung	SCC	Nivolumab	24.4
14	3.2	3 × 12.5 Gy at 65%	–	4.8		N1, N2, N3	4.8	Contr. lung	–	–	19.3
15	20.6	8 × 7.5 Gy at 80%	88.3	–		–	40.8	Contr. lung, adr., pleu., bone	ACC	Pemetrexed	92.5
16	8.4	8 × 7.5 Gy at 80%	8.3	8.3		N1, N2	8.3	Pleu.	SCLC	Carboplatin and etoposide	13.3
17	7.0	8 × 7.5 Gy at 80%	–	13.5		N1	14.0	CNS	–	–	17.2
18*	13.2	8 × 7.5 Gy at 80%	–	9.1		Ipsi. lung	–	–	–	–	123.3
19*	1.9	8 × 7.5 Gy at 80%	–	2.8		Ipsi. lung	–	–	–	–	35.6

ACC, adenocarcinoma; adr., adrenal; CNS, central nervous system; contr., contralateral; DF, distant failure; Gy, Gray; ipsi., ipsilateral; LCC, large cell carcinoma; LF, local failure; mo, months; m, multiple; Nx, lymph node levels (TNM system); OS, overall survival; peritoneal, peritoneal metastasis; pleu., pleural metastasis; RF, regional failure; SCLC, small cell lung cancer; SCC, squamous cell carcinoma; SCV, supraclavicular lymph nodes; UC, urothelial carcinoma.

*These six patients received a second course of radiotherapy for an intrapulmonary recurrence (see **Table 1B**).

**This patient was diagnosed with laryngeal carcinoma approximately 6 months after SBRT, received radiation therapy for that diagnosis, and was later treated with nivolumab following mediastinal and pulmonary progression.

**Table 1B:** Individualized characteristics and outcome of patients undergoing a second course of radiation.

Patient no.	Target volume for 2nd RT relative to PT	Tumor volume [cc]	Time to second radiation (mo)	Dose fractionation	Time to LF (mo)	Time to RF (mo)	Location of regional failure	Time to DF (mo)	DF location
2	LF	11.5	7.5	3 × 15 Gy at 65%	–	6.2	N1	–	–
3***	Ipsilateral lung	21.4	12.0	10 × 5 Gy at 80%	–	13.1	N1, 2, 3 (m)	–	–
9	Contr. lung	3.5	21.3	3 × 15 Gy at 65%	–	32.9	Ipsi. lung	–	–
10	Contr. lung	1.8	6.1	3 × 15 Gy at 65%	–	–	–	–	–
18***	Ipsi. lung	15.4	29.8	3 × 13.5 Gy at 65%	–	53.7	Ipsi. lung	–	–
19	Ipsi. lung	1.9	7.7	8 × 7.5 Gy at 80%	–	–	–	–	–

Contr., contralateral; DF, distant failure; Gy, Gray; ipsi., ipsilateral; LF, local failure; m, multiple; Nx, lymph node levels (TNM system); RF, regional failure; RT, radiotherapy.

***These patients received a third course of radiotherapy due to further intrapulmonary progression.

### Clinical outcome

**Figure 2:**
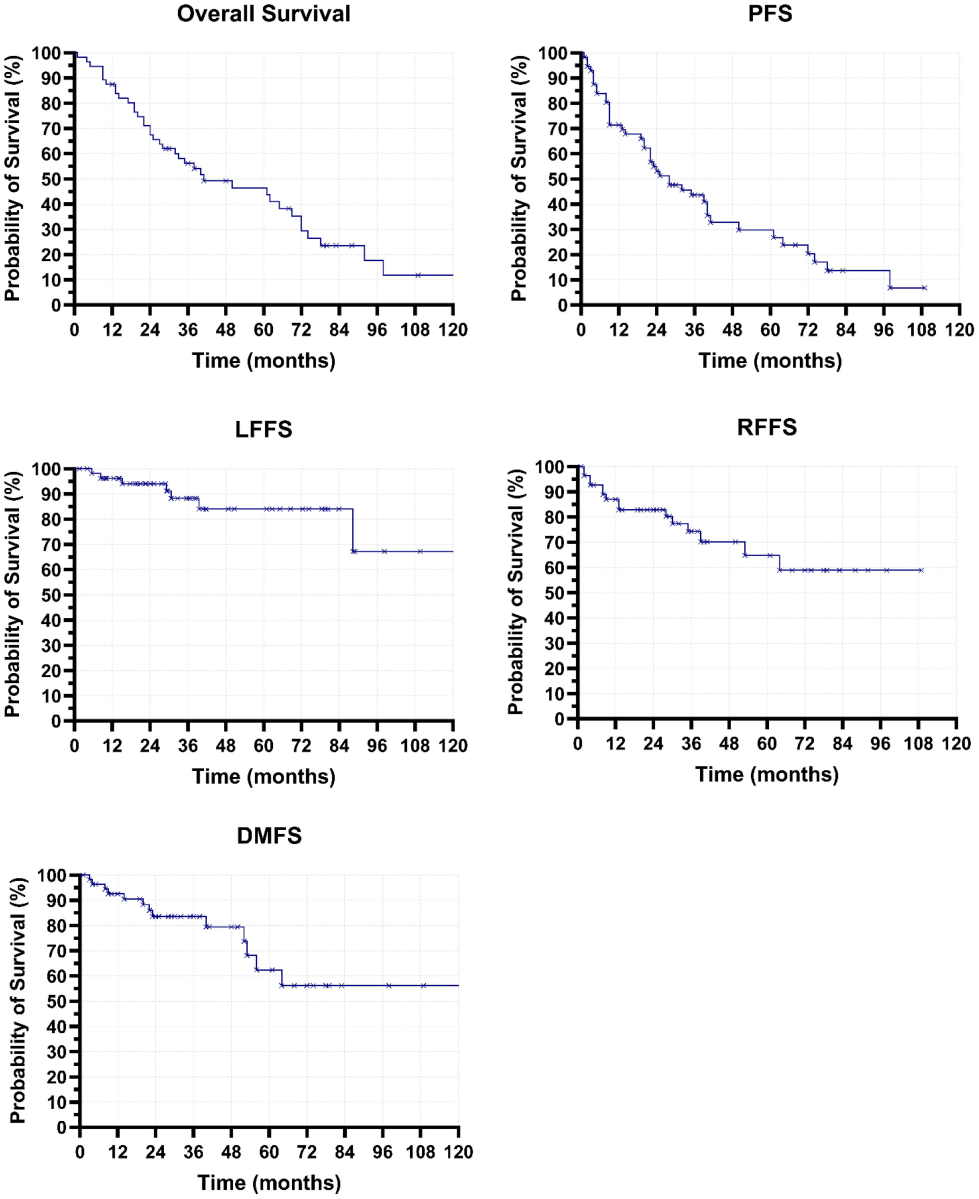
Kaplan–Meier estimates of overall survival (OS), progression-free survival (PFS), local failure-free survival (LFFS), regional failure-free survival (RFFS), and distant metastasis-free survival (DMFS).

With a median follow-up of 80.4 months (95% CI: 65.2–95.6) as of August 2025, 17 patients (30.4%) were still alive. Recurrence was observed in 19 patients (33.9%). Regional failure occurred in 15 patients (26.8%), and distant metastases were seen in 13 patients (23.2%). Intrapulmonary recurrence involved the ipsilateral lung in six patients (10.7%) and the contralateral lung in six patients (10.7%). Median PFS was 28.5 months (95% CI: 16.4–40.8), and median OS was 41.7 months (95% CI: 14.0–69.4). LFFS was 84.1% at 5 years and 67.3% at 10 years. RFFS was 64.7% at 5 years and 58.8% at 10 years. DMFS was 62.4% at 5 years and 56.1% at 10 years (**Figure 2**).

### Pathology of recurrence

Pathologic confirmation of recurrence was obtained in 10 of the 19 patients with any recurrence (52.6%). Among these, three patients (15.8%) were diagnosed with small cell lung cancer (SCLC), two (10.5%) with adenocarcinoma, two (10.5%) with squamous cell carcinoma (SCC), two (10.5%) with large cell carcinoma, and one patient (5.3%) with metastatic urothelial carcinoma. The last patient had a known history of bladder cancer, diagnosed 5.5 years prior to the development of the lung lesion.

For patients with histologically confirmed recurrence, the median overall survival was 33.0 months (95% CI: 17.2–48.9). The three patients with SCLC received chemotherapy and lived for 10.2, 13.3, and 65.2 months. One patient, later confirmed to have metastases from a previously diagnosed urothelial carcinoma, developed liver and multiple bone metastases on the first follow-up CT scan after SBRT.

### Pattern of first failure

**Figure 3:**
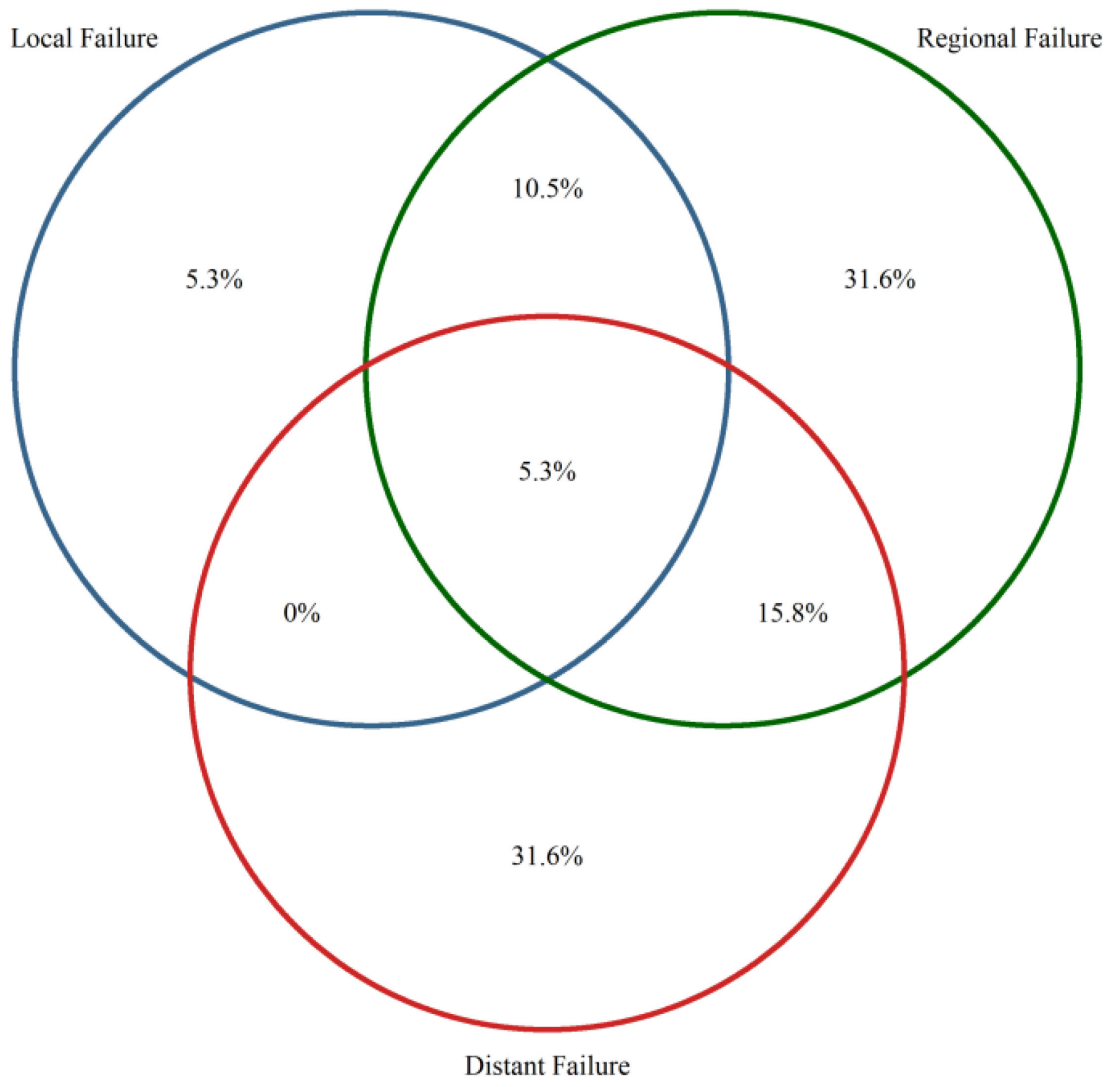
Patterns of first relapse following stereotactic body radiation therapy (SBRT). Venn diagram illustrating the distribution and overlap of recurrence sites, color-coded by frequency, with red indicating the most frequent and blue the least frequent recurrence patterns; values represent the percentage of patients within each failure category or overlapping combination.

Regional failure occurred in 13 patients (68.4%) as the first manifestation of recurrence, while distant metastases were observed in 11 patients (57.9%). Most regional recurrences involved intrapulmonary sites, affecting seven patients (36.8%). The most common pattern of distant metastasis was contralateral intrapulmonary spread seen in six patients (31.6%). **Table 2** and **Figure 3** present the distribution and frequency of first failure patterns following SBRT.

**Table 2:** Pattern of first failure after empiric SBRT.

Total patients with any failure	19 patients (100%)
Location distribution of first recurrence	
Local failure only	1 (5.3%)
Regional failure only	6 (31.6%)
Distant failure only	6 (31.6%)
Local and regional failure	2 (10.5%)
Regional and distant failure	3 (15.8%)
Local, regional, and distant failure	1 (5.3%)
Local failure as first manifestation	4 (21.1%)
Regional failure as first manifestation	12 (63.2%)
Ipsilateral hilar (N1)	7 (36.8%)
Mediastinal (N2)	6 (31.6%)
Contralateral hilar (N3)	4 (21.1%)
Supraclavicular (N3)	2 (10.5%)
Ipsilateral hung	5 (26.3%)
Distant failure as first manifestation	10 (52.6%)
Contralateral lung	6 (31.6%)
Bone	3 (15.8%)
Liver	2 (10.5%)
CNS	2 (10.5%)
Pleural	2 (10.5%)
Skin	2 (10.5%)
Peritoneal	1 (5.3%)
Adrenal	1 (5.3%)

SBRT, stereotactic body radiation therapy; CNS, central nervous system.

### Time to failure

Local failures after SBRT generally occurred earlier in the disease course, whereas distant metastases occurred later. The median time to local failure was 8.3 months (95% CI: 4.9–88.4), the median time to regional failure was 13.5 months (95% CI: 2.5–64.3), and the median time to distant metastases was 22.8 months (95% CI: 3.2–64.6).

### Competing risk analysis

**Figure 4:**
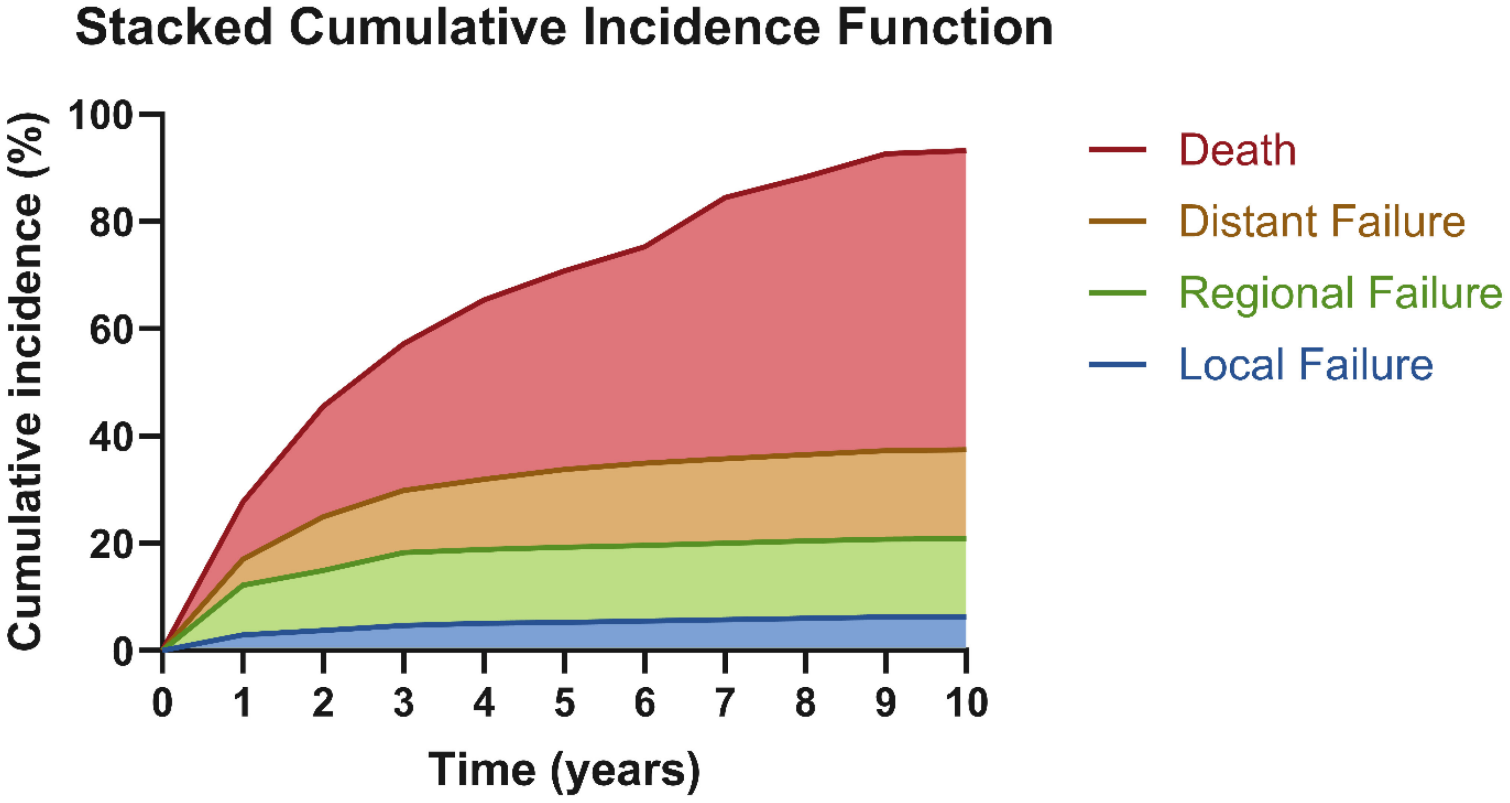
Estimated competing risk of recurrence or death over 10 years. This diagram compares the chances of different outcomes happening first during follow-up: local progression (blue), regional recurrence (green), distant recurrence (yellow), or death without recurrence (red). The risks of local, regional, and distant recurrences increased only slowly over time and reached a plateau after years. In contrast, the risk of death from other causes rose steadily and was the most common outcome in this group of patients who were mostly frail with multiple comorbidities.

The cumulative incidence of any event increased from 28.6% at 1 year to 56.3% at 3 years, 70.2% at 5 years, and 93.2% at 10 years. Local failure remained low and plateaued early: 3.6% at 3 years (95% CI: 0.0%–8.5%) and 6.3% at 5 to 10 years (95% CI: 0.0%-13.5%). Regional failure reached 14.6% by 3 to 5 years (95% CI: 5.1%–24.1%) and then remained stable through 10 years. Distant failure continued to rise after 3 years, increasing from 10.9% at 3 years (95% CI: 2.5%–19.2%) to 16.6% at 6 years (95% CI: 5.5%–27.6%) and then remaining stable through 10 years. Death continued to accumulate across follow-up—10.7% at 1 year (95% CI: 2.5%–18.9%), 35.8% at 5 years (95% CI: 21.8%–49.8%), and 55.8% at 10 years (95% CI: 36.6%–74.9%)—and dominated late outcomes (**Figure 4**).

The mean predicted probability of malignancy (Mayo Clinic SPN Malignancy Risk Score) for the entire cohort (n = 56) was 69.0% (SD 28.3%; range, 2.6%–97.5%). According to the established stratification criteria, three (5.4%) nodules were classified as low risk, 11 (19.6%) as intermediate risk, and 42 (75.0%) as high risk.

### Predictors of patient outcomes

No statistically significant associations were observed between GTV, BED, age, or tumor diameter and any of the evaluated endpoints, including OS, PFS, LFFS, RFFS, or DMFS (all p > 0.05).

### Subsequent curative-intent treatment

Seven patients received additional curative treatments. One underwent surgical resection for a solitary contralateral pulmonary metastasis, and six with solitary intrapulmonary recurrence received a second course of SBRT. Among these, two were treated for marginal recurrences (at the radiation field margin), two for new solitary ipsilateral lesions, and two for new solitary contralateral metastases. Overall survival for the five patients with solitary pulmonary nodule progression treated with curative intent was 35.6, 48.8, 80.4, 88.9, and 123.3 months, while the patient who underwent surgical resection for a new lesion had an overall survival of 92.5 months. Two patients ultimately received a third course of radiotherapy.

## Discussion

Our analysis confirms that empiric SBRT offers excellent local tumor control in presumed early-stage lung cancer. Seven of 56 treated patients (12.5%) experienced an in-field (local) failure, consistent with the literature, where 3–5-year local control rates commonly exceed 90% after SBRT for stage I NSCLC (25–27). Regional failures occurred in 15 patients (26.8%) and distant metastases in 13 (23.2%). This pattern shows that although SBRT reliably sterilizes the treated tumor, occult micro metastatic disease often determines the final outcomes.

Our findings align with prior reports that regional and distant metastases are the predominant sites of failure after curative SBRT (25, 26, 28). The higher rate of regional failures in our cohort probably reflects our classification method, which considered ipsilateral lung recurrences as regional events. In contrast, other studies often treat such lesions separately or view them as new primary tumors, especially in heavy smokers, who are at increased risk of developing multiple primary malignancies (27, 28). Given the substantial smoking history in our cohort (median 50 py), this interpretation seems clinically reasonable, as patients with solitary intrapulmonary recurrence who underwent a second curative-intent treatment, either surgical resection or SBRT, achieved favorable long-term outcomes, highlighting the value of additional curative approaches in this setting.

Histology appears to influence recurrence patterns in NSCLC, with studies reporting greater microscopic extension in adenocarcinoma than in SCC (29, 30). Reinhardt et al. further suggested that some local failures may result from insufficient dose coverage of these regions (31). Despite such findings, current SBRT guidelines recommend direct GTV-expantion to Planning Target Volume (PTV) without a defined clinical target volume (CTV) (32). In our cohort, the sample size was too small to detect a significant association between histology and outcomes.

Competing risk analysis demonstrated that non-cancer-related mortality represents a major limitation to long-term survival in this medically frail cohort, with the 10-year cumulative incidence of death without prior recurrence approaching 55%. In contrast, local failures were infrequent and occurred early, with a median time to recurrence of 8.3 months, compared with 13.5 months for regional and 22.8 months for distant progression.

The competing risk analysis shows that local failures were infrequent and plateaued early (≤5 years), supporting the durability of SBRT for local control. In contrast, distant failure continued to accumulate after 3 years, reaching 16.6% by year 6, signifying a later window of systemic relapse. Death accumulated over the follow-up period, and by 10 years, it was the leading outcome, emphasizing the importance of managing long-term comorbidities alongside cancer control in this frail, elderly group.

In a study comparing surgical resection and SBRT for early-stage NSCLC, patients who underwent surgery showed superior regional control (82.9% *vs*. 78.1%; p = 0.912), distant control (76.1% *vs*. 54.0%; p = 0.152), and cancer-specific survival (81.3% *vs*. 75.3%; p = 0.923), although none of these differences reached statistical significance (33). Comparable findings were reported in a cohort study of 9,001 patients who underwent surgical resection for early-stage NSCLC, in which 21.5% experienced distant recurrence within 5 years of treatment. Within this surgically eligible population, older age was associated with a significantly reduced likelihood of receiving treatment after recurrence, with an odds ratio of 0.42 for patients aged ≥75 years compared with those aged <55 years. Likewise, the presence of significant comorbidities was associated with lower rates of active treatment following the development of distant metastases (34). In our cohort, 10 patients underwent biopsy confirmation after recurrence, and among the 19 patients who experienced recurrence, six did not receive any subsequent therapy for their disease.

The combination of SBRT and checkpoint inhibitors has attracted considerable attention following the demonstrated survival benefit of immunotherapy in locally advanced and metastatic NSCLC (35–37). Chang et al. conducted a phase II trial in patients with early-stage NSCLC and isolated intrapulmonary recurrence, comparing SBRT alone with SBRT combined with nivolumab. In the SBRT arm, the rates of first relapse were 13.3% local, 10.7% regional, and 16.0% distant, whereas in the SBRT plus immunotherapy (I-SABR) arm, the corresponding rates were 0%, 6.1%, and 3.0%, respectively. The addition of immunotherapy reduced the overall recurrence rate from 36.0% to 12.1% (8). However, initial results from phase III trials have yet to corroborate these findings. A study comparing SBRT alone with SBRT plus concurrent and adjuvant atezolizumab demonstrated no survival benefit. Interestingly, the combination arm showed a higher local recurrence rate (13%) compared with SBRT alone (7%) (38). Results from two additional ongoing trials, PACIFIC-4/RTOG 3515 and KEYNOTE-867, which are assessing SBRT combined with durvalumab and pembrolizumab, respectively, are expected to provide further insights into the efficacy and safety of these approaches (39, 40).

### Limitations

This retrospective single-center study is subject to several limitations. First, the limited sample size, particularly the subgroup of patients who experienced recurrence, constrains the statistical robustness of subgroup analyses and limits the generalizability of the findings. Second, the absence of histopathological confirmation in all cases of recurrence restricts the ability to draw definitive conclusions regarding underlying tumor biology and recurrence patterns. The inclusion of such data would have yielded more comprehensive insights. Third, due to the frailty and comorbidities of the patient population, including chronic obstructive pulmonary disease (COPD), uniform administration of ablative radiation doses was not feasible. Consequently, heterogeneous SBRT fractionation regimens were employed, which may have influenced treatment outcomes, thereby further limiting the applicability of the results to broader clinical settings.

## Conclusion

Empiric SBRT for presumed early-stage NSCLC achieved excellent and durable local control, but long-term outcomes were constrained by comorbid mortality and regional or systemic progression. Importantly, selected patients with solitary pulmonary nodule recurrence achieved durable survival with additional curative-intent therapy, emphasizing the need for individualized follow-up and treatment strategies. Overall, these findings highlight both the effectiveness of SBRT in tumor sterilization and the necessity of prolonged surveillance and tailored systemic approaches to mitigate late metastatic progression in high-risk patients.

## Data Availability

The original contributions presented in the study are included in the article/supplementary material. Further inquiries can be directed to the corresponding author.
